# The London Major Trauma Network System: A Literature Review

**DOI:** 10.7759/cureus.12000

**Published:** 2020-12-09

**Authors:** Mohammad Waseem Beeharry, Komal Moqeem

**Affiliations:** 1 Trauma and Orthopaedics, Barts Health NHS Trust, London, GBR; 2 Internal Medicine, Royal Surrey County Hospital, Guildford, GBR

**Keywords:** major trauma centre, trauma unit, london trauma system, inclusive trauma network system

## Abstract

Trauma is one of the leading causes of death and disability worldwide and is a major global public health problem. The provision of trauma care has been substandard in England and Wales prior to the implementation of an inclusive major trauma network system in London in 2010 and subsequently across the rest of England two years later. The implementation of the London trauma system has brought about improvements to the delivery of trauma care by decreasing the overall morbidity and mortality significantly. This framework encompasses the collaboration of emergency services, designated Major Trauma Centres (MTCs), Trauma Units (TUs) and community providers which have been optimized with the expertise and resources to provide the best outcomes for major trauma patients. Specific triage protocols, consultant-led trauma service and on-the-spot access to radiology services and operating theatres have played a pivotal role in the improvement of trauma care. In spite of several strengths, however, the London major trauma network system is by no means without its limitations. The emergence of the new coronavirus disease 2019 (COVID-19) pandemic has created major barriers to the smooth running of trauma services by exhausting resources due to infection control measures, reduced theatre space and re-deployment of medical staffs. In addition, the cancellation of elective surgeries has impacted directly on the training of surgical trainees by leaving them with significantly reduced surgical exposure. As a results of this ever changing surgical landscape, a need to urgently review these traditional surgical training methods with a view to modernize the curriculum. Although the London trauma system has evolved significantly since its implementation, its limitations should be recognized and addressed to enhance performance and improve patient outcomes.

## Introduction and background

Trauma is one of the leading causes of death and disability worldwide and is a major global public health problem. It is estimated that about 5.8 million people die each year as a result of injuries, accounting for 10% of deaths worldwide [1]. In England and Wales alone, trauma accounts for over 16,000 deaths each year [2]. 

A report of the National Confidential Enquiry into Patient Outcome and Death (NCEPOD) in 2007 showed that almost 60% of major trauma patients across England, Wales, Northern Ireland and Offshore islands had received a standard of care that was less than good practice. Several deficiencies in many areas in the provision of care was identified such as organisation of pre-hospital care, trauma team response, lack of seniority and experience of staffs involved amongst others [3]. 

In order to address this issue and reduce trauma-related mortality and disability rates, an inclusive trauma network system was implemented in London in 2010 and the rest of England for two years. The London trauma framework encompasses four trauma networks (North West London, North East London and Essex, South West London and Surrey, South East London, and Kent trauma networks). Each of these networks is led by a Major Trauma Centre (MTC) to treat the most critically wounded patients, and consequently, each MTC is associated with other local Trauma Units (TU) for patients with less serious injuries. 

## Review

Strengths

Implementation of the regional London trauma system with the overall aim of reducing morbidity and mortality has been associated with significant positive changes. Patients hospitalized with serious injuries and treated within an inclusive trauma system have a 23% lower mortality rate compared to those treated in regions with few high-level trauma centres (exclusive trauma system) [4]. 

Specific triage protocols have been designed and introduced to help facilitate decision making in transferring patients to an MTC based on several factors such as the mechanism of injury, assessment of patient physiology, individual patient factors, and anatomy of apparent injuries [5]. 

The fundamental aspect which makes this system function is the close collaboration and coordinated effort between emergency services, MTCs and TUs, and community providers. This helps deliver the full range of care to the trauma patient across the entire pathway from the point of injury until rehabilitation. 

Progressive improvements in patient outcomes have been identified by the Trauma Audit and Research Network (TARN), showing the advantages of such networks [6]. A more recent study carried out by Cole et al. (2016) to evaluate the impact of the London trauma system on quality of care and patient outcomes have demonstrated substantial improvements in the overall quality of care, and these were mainly seen in MTCs. These improvements, associated with increased early survival, were seen primarily due to an enhancement in organizational processes such as the prompt delivery of specialist multi-disciplinary care, significant trauma team response in the initial assessment of the severely injured patient, and immediate access to radiological modalities (including interventional radiology) and operating theatres [7]. 

MTCs have been optimized with the expertise and resources to provide the best outcomes for major trauma patients. They have dedicated trauma teams and provides emergency access to consultant-led trauma service with multi-specialty care (such as general, emergency medicine, vascular, trauma and orthopaedics, plastic, spinal, maxillofacial, cardiothoracic and neurological surgery, critical care, and interventional radiology) around the clock. 

The need for clear and effective clinical leadership, as discussed by Tiel Groenestege-Kreb et al. (2014), has shown to be of particular importance in the management of the trauma patient. The designation of a dedicated consultant-led trauma team approach allows for quicker evaluation and resuscitation, which can lead to a decrease in time from injury to vital interventions [8]. Moreover, they state that the availability of an attending trauma surgeon on the trauma team 24 h a day reduces resuscitation time and time to the incision for emergency operations. This has been supported by a prospective analysis done by Driscoll and Vincent of 207 trauma patients from three internationally recognized trauma centres, which has shown that resuscitation was reduced by over half from 122 to 56 min when carried out by a dedicated trauma team [9]. 

The current trauma provision in London has not only provided multi-disciplinary specialist care in a timely fashion but has also been complemented by rapid access to investigations and interventions. Since the overhaul of trauma services, on-the-spot access to radiology services (diagnostic and interventional) has played a key role in the rapid diagnosis, effective triage, and management of complex polytrauma patients [10]. Immediate 24/7 expert imaging guidance by a resident radiologist in combination with radiological modalities such as Trauma CT scans has become a vital aspect of trauma care in the early detection of life-threatening injuries, with an estimated 45% of CT scans performed within 30 minutes of arrival [11]. 

The beneficial aspect of this is that not every hospital within the regional network requires their own 24/7 service, but rather formal arrangements and protocols in place between MTCs and TUs allow access to this service when required between hospitals. Furthermore, MTCs and TUs are linked via an Image Exchange Portal (IEP) system, enabling quick transfer of radiological images where secondary advice can be sought. Within the London trauma network, over 5,000 images are exchanged per month between hospital networks using the IEP system, saving both time and decreasing costs [12]. 

Overall, MTCs have resulted in a high load of trauma patients to be managed in one centre, improving staff expertise and skill. This, in conjunction with effective triage, consultant-led service provision, and concentration of resources to where it is required most, has improved the care of the trauma patient across the network.

Limitations

Although the implementation of the major trauma network in London has provided robust improvement in the management of the trauma patient in terms of improving patient outcomes, it is by no means without other major challenges. 

The emergence of the new coronavirus (COVID-19) pandemic has posed substantial obstacles to the smooth running of trauma services across the London trauma network. Trauma services have had to be re-configured due to considerable pressures placed on the network in order to facilitate the provision of medical care to the COVID-19 patient at the expense of a full functional trauma service [13]. Although trauma care remained a priority, several changes were implemented as a result of infrastructure demand from COVID-19, infection control measures, and staff absence due to either re-deployment or illness. This has necessitated rapid development and implementation of hospital guidelines and early adaptive strategies depending on the available resources and local infrastructure across major trauma centres and trauma units [14]. 

One of the major impacts of the current pandemic was the increasing constraint placed on the provision of surgery (both trauma and elective). In view of anaesthetic staff re-deployment and alteration of operating rooms as an extension to critical care, elective surgeries were postponed, and the threshold for trauma surgeries re-considered [13]. Recommended guidelines from the British Orthopaedic Association Standards for Trauma and Orthopaedics (BOAST) have advocated the conservative management of most upper limb fractures (including humeral, clavicle, and wrist fractures) with known high rates of union [15]. However, the drawback of this non-operative approach is a potential late displacement of fractures resulting in malunions or non-unions, which would subsequently require operative management at a later date [13]. 

Furthermore, the cancellation of elective surgeries has had a substantial impact on training, leaving surgical trainees in a vulnerable position with significantly reduced surgical exposure. The smooth running of the trauma network relies heavily on the education and training of future specialist care providers, mainly surgical trainees. The surgical training program in the UK is a competency-based curriculum that allows trainees to progress according to their ability and achievement. 

Within an inclusive system such as the London Trauma Network system, where there are more dispersed populations and geography, TUs within the network plays a vital role by providing the vast majority of trauma care as per the trauma network initiative [16]. However, the exact re-distribution of trauma patients across the London network is unclear and therefore raises questions about the extent of surgical trainee exposure to trauma cases. A case study carried out by Hipps et al. (2015) for the Northern Trauma Network across eight TUs and two MTCs, has shown that since the implementation of their trauma network, indexed trauma procedures such as intramedullary nailing procedures have reduced by 16% at TUs but have risen by 19% at their two MTCs. Although they’ve seen a reduction in exposure to indexed operations at both MTCs and TUs, trainees at MTCs gained greater operative experience in trauma procedures associated with high energy such as intramedullary nailing and were also performing 10% more trauma operations than their counterparts at TUs [16]. Since the knowledge and skills of surgeons are critical for surgical success, this, therefore, poses a dilemma for the surgical training program to provide high-quality training. The possibility of alternate and standardized approaches to training might be increasingly necessary.

Recommendations

The ideal trauma network would be an inclusive trauma-related service system whereby all acute hospitals within the region are able to participate and provide different levels of service to the extent that their resources allow. The basis of most trauma systems components remains the same integrated approach to trauma - prevention, pre-hospital care, and hospital care through rehabilitation [5]. 

One of the main priorities in any trauma system would be to develop and implement an effective triage model and referral framework, according to the levelling and capacities of MTCs and TUs within the region. The main aim of an effective triage model with triage protocols would be to ensure that the right individual is taken to the right place at the right time in order to receive definitive trauma management. 

The assessment of trauma patients at the scene by emergency services remains a vital aspect and integral component of the trauma system. This helps determine if the patient’s injuries would be better supported by transferring them to an MTC directly rather than a TU. Effective triaging can prevent delay in the definitive management of the major trauma patient by transferring them directly to an MTC, and this has been associated with better patient outcomes. On the other hand, if triaging is not done properly (over triaging), such as when a patient with relatively minor injuries is transferred directly to an MTC, can exhaust the system's resources and potentially compromising the treatment of other patients [6]. A study carried out by Newgard et al. (2014) estimated that a 30% over-triage rate is potentially costing trauma systems an excess of $136 million annually in the USA [17]. 

The National Health Service (NHS) Clinical Advisory Groups (NCAG) has advised the stratification of the trauma patient at the point of injury, based on the World Health Organisation (WHO) and American College of Surgeons (ACS) committee guidelines, on four elements: physiological derangement, anatomical injury, mechanism of injury and special circumstances. However, this particular version had initially led to significant over-triage rates in the London trauma system, where only 317 (29 %) of 1,088 patients who triggered the tool were found to have an Injury Severity Score (ISS) >15 in that system [18]. 

Nevertheless, owing to the complex physiological responses of patients to trauma, triage is very often inexact. Some patients may have a delayed physiological response to trauma, whereas other patients sustaining minor injuries can result in morbidity and mortality as a result of their age or co-morbidities [19]. A literature review carried by Mackenzie et al. (2013) demonstrated that physiological derangement (such as Glasgow Coma Scale [GCS]) and anatomical injury (such as pelvic and skull fractures) were the most relevant parameters for accurate triage decisions [18]. 

One of the other main priorities in developing a major trauma system would be placing great emphasis on the training of the trauma surgeon of the future. Investment in the training needs of not only surgical trainees but also anaesthetic and emergency medicine trainees is one of the critical aspects of the long successful run of a trauma system. 

The surgical landscape in recent years has been changing, bringing with it additional pressures on training even before the COVID-19 pandemic. This has posed a major dilemma for surgical trainees and trainers in the United Kingdom (UK) as to how adequate surgical training and experience can be sustained, given the reduction of theatre exposure time. This has inadvertently caused an adverse impact on training. This calls for a need to urgently review these traditional surgical training methods with a view to modernize the curriculum. In a competency-based model such as the UK, with the need to achieve specific operative competencies and to continue professional development, a virtual reality platform (simulation training) and multimedia-based learning can offer an intriguing solution as a learning modality in a completely safe, robust, and realistic training/teaching environment [20]. Hence, a modernised approach with alternate and standardized methods to teaching and training is increasingly necessary, given the ever-changing landscape of COVID-19. 

## Conclusions

The trauma system within London has evolved significantly since its implementation in 2010 and continues to do so. There is no doubt that the trauma-related healthcare community continues to strive for a trauma system framework that enhances performance and improves patient outcomes. However, the limitations of MTCs need to be recognized. Whilst it improves patient outcomes by concentrating skills and resources in one centre, it risks de-skilling and limited exposure of surgical trainees working in non-MTCs. The new trauma network has embedded itself as a core part of the management of the critically wounded patients and is here to stay. This, coupled with the unprecedented effects COVID-19 has brought to the surgical landscape, urgently calls for a review of a surgical training curriculum to ensure each surgical trainee has adequate trauma exposure and develop a robust skill set in managing trauma patients. 
